# The Impact of Low-Density Lipoprotein Equation Changes on Cholesterol Treatment in Canada

**DOI:** 10.1016/j.cjco.2022.09.007

**Published:** 2022-10-01

**Authors:** Pei Jun Zhao, Robert A. Hegele

**Affiliations:** a Division of Cardiology, Department of Medicine, Schulich School of Medicine and Dentistry, Western University, London, Ontario, Canada; b Division of Endocrinology, Department of Medicine, Schulich School of Medicine and Dentistry, Western University, London, Ontario, Canada; c Robarts Research Institute, Western University, London, Ontario, Canada

## Abstract

**Background:**

In cardiovascular disease prevention, low-density lipoprotein cholesterol (LDL-C) values guide treatment for lowering cholesterol level. After 50 years of clinical laboratories using the Friedewald LDL-C equation, the Canadian Society of Clinical Chemists recently recommended adoption of the new and more accurate Sampson / U.S. National Institutes of Health (NIH) LDL-C equation. Here, we estimate the anticipated population-level impact of this equation change.

**Methods:**

We compared lipid profiles from the Canadian Health Measures Survey (CHMS) year 2019 to those from the National Health and Nutrition Examination Survey (NHANES) years 2017 to 2020. Then, based on 10,828 participants in the latter, we calculated the impact of changing the LDL-C equation from the Friedewald to the Sampson.

**Results:**

Sampson- and Friedewald-equation LDL-C values are strongly correlated (*r* = 0.99, *P* < 0.001), but differences between them increase with both higher triglyceride and lower LDL-C values. We evaluated the impact of these discordances using LDL-C treatment thresholds from the 2021 Canadian Cardiovascular Society lipid guidelines. Among patients who take cholesterol-lowering medications, the Sampson equation reclassifies 3.3% more patients (95% confidence interval 2.2% to 4.9%), or about 123,000 individuals, as meeting the criteria for treatment intensification.

**Conclusion:**

Although changing the LDL-C equation used from the Friedewald to the Sampson affects only a small proportion of the population, an estimated 123,000 Canadians who are taking cholesterol-lowering medications may need to intensify treatment to lower their cholesterol level, due to small absolute changes around guideline threshold values of LDL-C.

## **Résumé**

**Contexte:**

Dans la prévention des maladies cardiovasculaires, les valeurs de cholestérol à lipoprotéines de faible densité (cholestérol LDL) guident le traitement hypocholestérolémiant. Depuis 50 ans, la formule de Friedewald est utilisée par les biochimistes pour calculer le cholestérol LDL. Or, la Société canadienne des clinico-chimistes vient de recommander l’adoption d’une nouvelle équation plus précise pour évaluer le cholestérol LDL : l’équation Sampson (National Institutes of Health des États-Unis). Nous analysons ici les conséquences que pourrait avoir cette nouvelle méthode de calcul à l’échelle de la population.

**Méthodologie:**

Nous avons comparé les bilans lipidiques issus de l’Enquête canadienne sur les mesures de la santé (ECMS) de 2019 à ceux de la National Health and Nutrition Examination Survey (NHANES) de 2017 à 2020. En nous appuyant sur les données de 10 828 participants à cette dernière enquête, nous avons évalué l’effet qu’aurait l’utilisation de la méthode de Sampson sur la décision thérapeutique.

**Résultats:**

Les valeurs de cholestérol LDL obtenues avec les équations de Sampson et de Friedewald sont fortement corrélées (*r* = 0,99, *P* < 0,001), mais l’écart se creuse lorsque le taux de triglycérides est plus élevé ou que le taux de cholestérol LDL est plus faible. Nous avons évalué l’effet de cette divergence en appliquant les valeurs seuils de traitement du cholestérol LDL établies par les lignes directrices de 2021 sur la dyslipidémie de la Société cardiovasculaire du Canada. Parmi les patients prenant des médicaments pour abaisser leur taux de cholestérol, l'équation de Sampson reclasse 3,3 % plus de patients (intervalle de confiance à 95 % : 2,2 % à 4,9 %), soit environ 123 000 personnes, comme répondant au critère d’intensification du traitement.

**Conclusion:**

L’utilisation de la méthode de calcul de Sampson plutôt que de la méthode de Friedewald a une incidence uniquement sur une frange marginale de la population. Néanmoins, c’est quelque 123 000 Canadiens sous hypocholestérolémiants qui pourraient devoir intensifier leur traitement si, en raison d’une variation absolue minime, leur taux de cholestérol LDL atteint les valeurs cibles de traitement.

Low-density lipoprotein cholesterol (LDL-C) level is strongly associated with risk of atherosclerotic cardiovascular disease (ASCVD).^1^ Thus, lowering the LDL-C level is a fundamental component of ASCVD treatment, in both primary and secondary prevention. In the laboratory, the gold-standard measurement of LDL-C values uses ultracentrifugation to separate lipoproteins by density, and the overall process called β-quantification directly yields an LDL-C result.^2^ However, this process is both time-consuming and expensive, and most laboratories are not capable of performing it. Instead, LDL-C values are typically estimated using variables that are easy to measure and do not require ultracentrifugation, namely total cholesterol (TC) level, high-density lipoprotein cholesterol (HDL-C) level, and triglyceride (TG) level.

In 1972, Friedewald empirically derived an LDL-C equation using β-quantification results in 448 individuals, which was approximately linear and was validated for TG ≤ 4.52 mmol/L.^2^ For decades, the Friedewald equation has been the standard for calculating LDL-C level, in Canada and around the world, as follows:

$$\text{LDL-C}=\text{TC}-\text{HDL-C}-\frac{\text{TG}}{2.2}\;\text{(Friedewald equation; units in mmol/L)}$$

Given that the ratio of cholesterol to TG molecules in the very-low-density lipoprotein fraction is highly variable, the LDL-C estimate deteriorates when TG levels are high. Because hypertriglyceridemia is increasing in prevalence in Westernized societies due to a greater prevalence of obesity and diabetes,^3^ the Friedewald equation is becoming less accurate in a higher proportion of people. Alternative LDL-C equations have been proposed over the years, mainly by developing different coefficients to terms in the Friedewald equation.^4^, ^5^, ^6^, ^7^ In addition, LDL-C equations based on the direct homogenous assay for LDL have been formulated.^8^, ^9^, ^10^ In 2013, Martin et al. at Johns Hopkins University derived an updated LDL-C equation using vertical-rotor ultracentrifugation (similar to β-quantification) in 900,605 individuals, which was validated for TG levels ≤ 4.52 mmol/L.^11^ This equation seemed to be an incremental improvement over the Friedewald equation, but it was never widely adopted in Canada, mainly for proprietary reasons. Subsequently, in 2021, an extended Martin/Hopkins equation was developed for individuals with TG level from 4.52 to 9.03 mmol/L,^12^ as follows:

$$\text{LDL-C}=\text{TC}-\text{HDL-C}-\frac{\text{TG}}{coefficient}\;\text{(Martin/Hopkins equation; units in mmol/L)}$$

where the *coefficient* is empirically derived and referenced in a 180-cell table depending on TG and non-HDL-C.

In 2020, using multiple least-squares regression, Sampson and colleagues, at the U.S. National Institutes of Health (NIH), derived a new LDL-C equation from β-quantification reference values in 8656 fasting patients, validated for TG levels ≤ 9.04 mmol/L and excluding patients with the rare diagnosis of dysbetalipoproteinemia (formerly called type III hyperlipoproteinemia).^13^ Currently, the Sampson/NIH equation has proven to be among the most accurate for calculating LDL-C level, as follows^14^, ^15^, ^16^, ^17^, ^18^:

$$\text{LDL-C}=\frac{\text{TC}}{0.948}-\frac{\text{HDL-C}}{0.971}-\left(\frac{\text{TG}}{3.74}+\frac{\text{TG}\times \text{Non-HDL-C}}{24.16}-\frac{{\text{TG}}^{2}}{79.36}\right)-0.244\;\text{(Sampson/NIH equation; units in mmol/L)}$$

In 2022, after 50 years of use of the Friedewald LDL-C equation, the Canadian Society of Clinical Chemists has officially recommended nationwide implementation of the Sampson/NIH equation^19^ Since July 2022, laboratories in Canada have started to switch from the Friedewald to the Sampson/NIH equation. Here, we evaluate the potential public health impact of changing from using the Friedewald to using the Sampson/NIH LDL-C equation.

## Methods

### Patient population

The Canadian Health Measures Survey (CHMS)^20^ and the US National Health and Nutrition Examination Survey (NHANES)^21^ consist of nationally representative population samples in Canada and the U.S., respectively. Although CHMS releases population summary statistics,^22^, ^23^, ^24^, ^25^, ^26^, ^27^ NHANES releases patient-level data. We first compared lipid profile distributions from CHMS year 2019 versus NHANES years 2017 to 2020. Then, using NHANES data, LDL-C was calculated by both the Friedewald and Sampson equations.

### Statistical analysis

To generate national estimates, survey analysis was performed, accounting for strata, primary sampling units, and participant weighting. Laboratory values were reported with mean and standard deviation, as well as with median and interquartile range. Population means were compared using both the paired *t*-test and the Wilcoxon signed-rank test. Variable distributions were compared using quantile-quantile plots. LDL-C kernel density was calculated by the Epanechnikov kernel function. Figures were produced in R 4.1.3 (R Foundation for Statistical Computing, Vienna, Austria) and Stata 15.1 (StataCorp, College Station, TX).

## Results

The 2019 CHMS includes 5797 participants, with ages ranging from 3 to 79 years. In the 2017 to 2020 NHANES cohort, 10,828 participants had their cholesterol level measured, of whom 9493 also had their TG level measured. Using survey analysis, population characteristics are listed in Table 1. In the CHMS study, the mean Friedewald LDL-C level was 2.7 mmol/L; 12% of participants were taking cholesterol-lowering medications (Table 2). In the NHANES study, the average age was 42.3 ± 18.8 years; 51.2% were female; and 15.8% were taking cholesterol-lowering medications. Fasting time before blood work averaged 9.3 ± 4.9 hours. The mean Friedewald and Sampson LDL-C levels were 2.72 ± 0.90 mmol/L and 2.78 ± 0.91 mmol/L, respectively, and the difference between them was highly statistically significant (*P* < 0.001). In CHMS and NHANES, the distributions of total cholesterol, HDL-C, TG, and Friedewald LDL-C were similar (Supplemental Fig. S1).

**Table 1:** Population characteristics of the National Health and Nutrition Examination Survey (NHANES) from 2017-2020 for individuals who had cholesterol data; estimates were derived from survey analysis and are reflective of the national population

Variable	All participants		On medication to lower cholesterol	
Age, y	42.3 ± 18.8	42 (25–59)	64.2 ± 10.5	65 (57–73)
Gender, female	51.2		48.6	
Ethnicity, %				
Non-Hispanic White	60.7		70.7	
Non-Hispanic Black	11.2		8.7	
Mexican American	8.0		6.1	
Other Hispanic	10.0		4.7	
Non-Hispanic Asian	5.6		5.4	
Other race, mixed race	4.5		4.5	
BMI, kg/m^2^	28.6 ± 6.9	27.6 (23.4–32.6)	30.9 ± 6.0	30.0 (26.4–34.5)
Hypertension, %	30.9		67.7	
Diabetes, %	9.8		33.1	
Coronary artery disease, %	4.2		16.8	
Stroke, %	3.6		10.4	
Congestive heart failure, %	2.6		7.5	
Hypercholesterolemia, %	32.9		87.1	
Taking cholesterol-lowering medications, %	15.8		100	
Fasting time, h	9.3 ± 4.9	10.1 (4.9–12.7)	9.3 ± 4.3	10.2 (4.9–12.8)
Total cholesterol, mmol/L	4.70 ± 0.95	4.60 (3.96–5.33)	4.47 ± 0.97	4.37 (3.75–5.09)
HDL-C, mmol/L	1.39 ± 0.36	1.32 (1.11–1.60)	1.36 ± 0.38	1.29 (1.06–1.58)
Triglyceride, mmol/L				
Fasting (≥ 8.5 h)	1.19 ± 0.95	0.96 (0.64–1.46)	1.46 ± 1.14	1.23 (0.81–1.75)
All	1.44 ± 0.99	1.16 (0.78–1.76)	1.72 ± 1.13	1.45 (0.96–2.08)
LDL-C, Friedewald, mmol/L	2.72 ± 0.90	2.66 (2.10–3.25)	2.35 ± 0.93	2.25 (1.70–2.87)
LDL-C, Sampson/NIH, mmol/L	2.78 ± 0.91	2.71 (2.14–3.32)	2.43 ± 0.94	2.33 (1.76–2.97)

Values are mean ± standard deviation, median (interquartile range), or %.

BMI, body mass index; HDL-C, high-density lipoprotein cholesterol; LDL-C, low-density lipoprotein cholesterol; NIH, (US) National Institutes of Health.

**Table 2:** Population characteristics of the Canadian Health Measures Survey (CHMS); estimates were derived from survey analysis and are reflective of the national population

Variable	CHMS 2019	
	Mean	Median (IQR)
Age, y, range	3 to 79	
BMI, kg/m^2^	26.0	25.3 (21.6–29.3)
Hypertension, %	22.4	
Hypercholesterolemia, %	28	
Taking cholesterol-lowering medications, %	12	
Total cholesterol, mmol/L	4.74	4.61 (3.97–5.38)
HDL-C, mmol/L	1.42	1.36 (1.12–1.64)
Triglyceride, fasting, mmol/L	1.08	1.01 (0.71–1.50)
LDL-C, Friedewald equation, mmol/L	2.7	2.5 (2.0–3.1)

BMI, body mass index; HDL-C, high-density lipoprotein cholesterol; LDL-C, low-density lipoprotein cholesterol.

**Figure 1:**
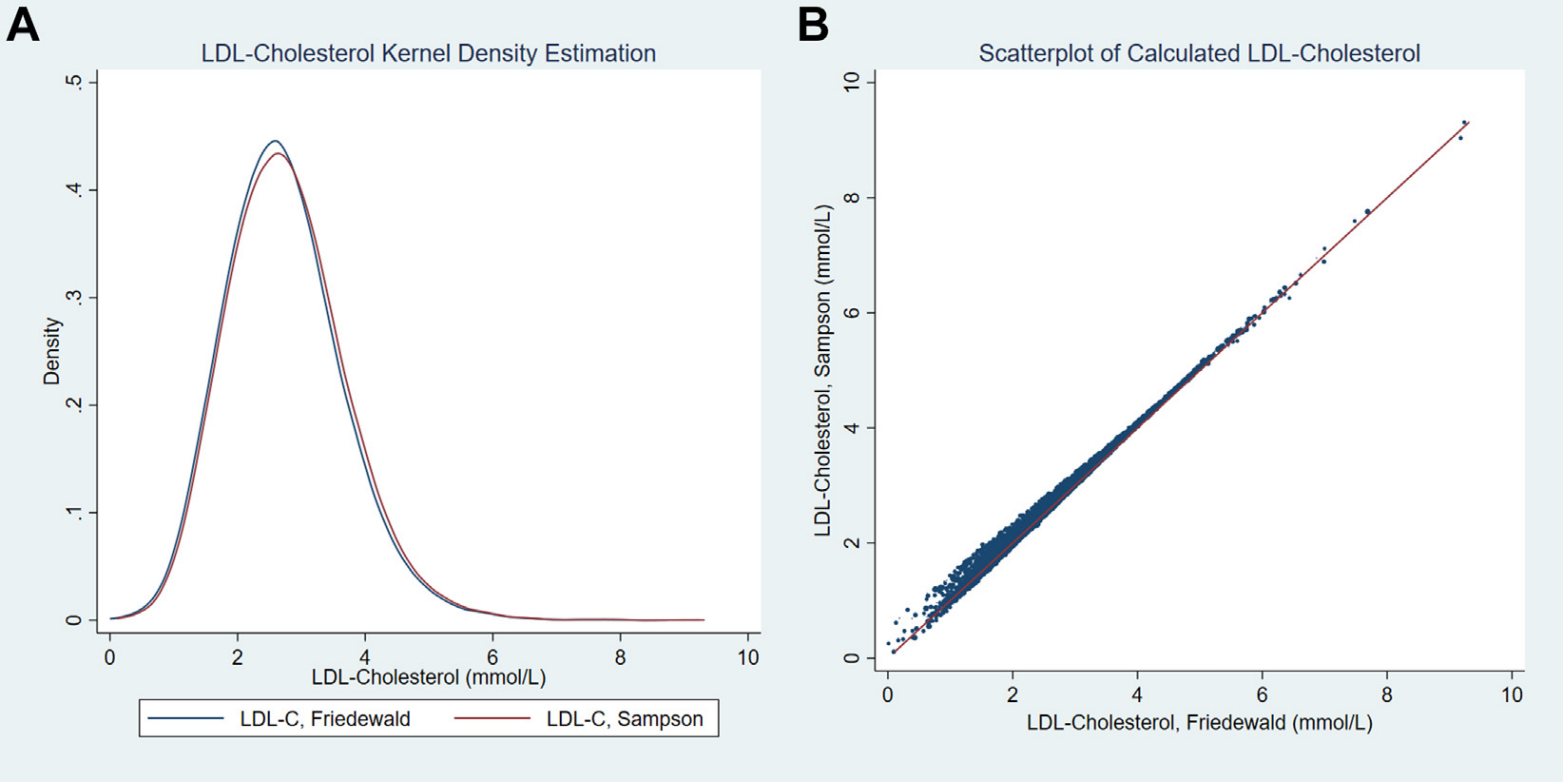
Comparison of the low-density lipoprotein cholesterol (LDL) values obtained by the Sampson vs Friedewald equations. (**A**) Kernel density estimation of LDL-cholesterol distribution in the National Health and Nutrition Examination Survey (NHANES) calculated by the Friedewald and Sampson equations. (Epanechnikov kernel function, bandwidth 0.2586.) Friedewald LDL-C mean 2.72 mmol/L, standard deviation 0.90, skewness 0.64; kurtosis 4.26; median 2.66, interquartile range 2.10-3.25. Sampson LDL-C mean 2.78 mmol/L, standard deviation 0.91, skewness 0.62; kurtosis 4.11; median 2.71, interquartile range 2.14-3.32. (**B**) Scatterplot comparing Sampson and Friedewald LDL-cholesterol values. Their Pearson correlation coefficient is 0.99. A Friedewald-equation LDL-C of 1.80 mmol/L corresponds to a Sampson-equation LDL-C of 1.86 mmol/L (95% confidence interval, 1.85 to 1.86 mmol/L).

**Figure 2:**
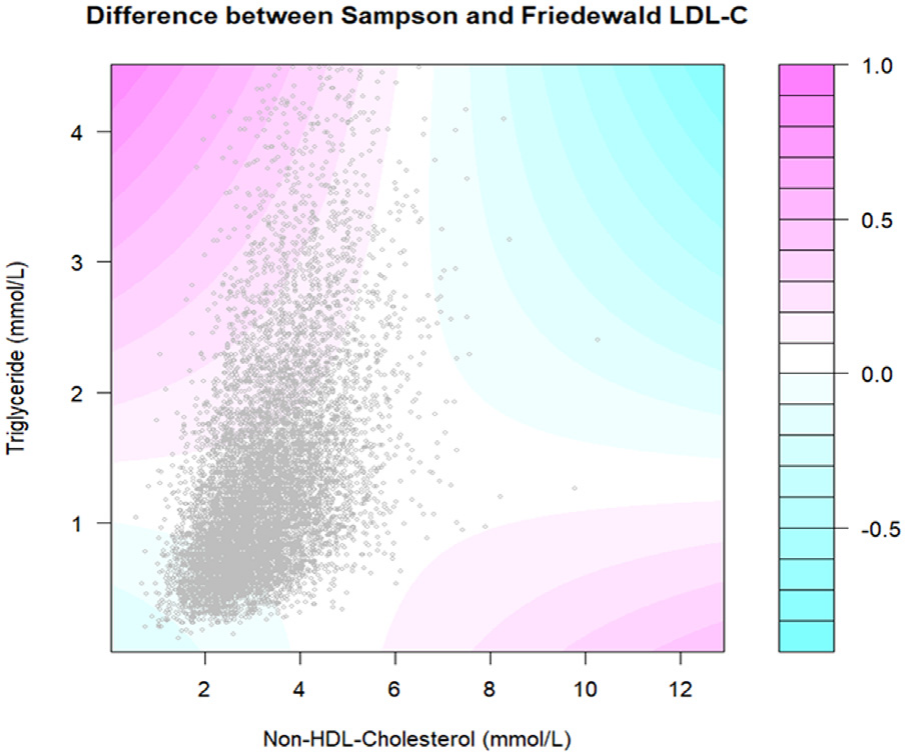
Contour map of difference between Sampson-equation and Friedewald-equation low-density lipoprotein cholesterol (LDL-C) in mmol/L by non-high-density lipoprotein (HDL)-cholesterol and triglyceride levels. The scatterplot consists of measurements from National Health and Nutrition Examination Survey (NHANES) participants. For most patients, the LDL-C difference is around 0 mmol/L. The contour curvatures reflect quadratic terms in the Sampson equation. The Sampson-equation LDL-C value is greater than the Friedewald-equation LDL-C value when non-HDL-C and triglyceride are discordant (for instance, high triglyceride and low non-HDL-C). Conversely, in a minority of cases, when non-HDL-C and triglyceride are concordant (for instance, low non-HDL-C and low triglyceride), the Sampson-equation LDL-C value is less than the Friedewald-equation LDL-C value.

Next, the national-level LDL-C distribution was estimated by kernel density (Fig. 1A). The Friedewald and Sampson LDL-C measures were well-correlated, with a Pearson coefficient of 0.99 (*P* < 0.001; Fig. 1B). Overall, the Sampson LDL-C value is slightly higher than the Friedewald LDL-C value. For instance, a Friedewald LDL-C value of 1.80 mmol/L corresponds to a Sampson LDL-C value of 1.86 mmol/L (95% confidence interval [CI] 1.85 to 1.86 mmol/L). When the Friedewald LDL-C value is less than 2.59 mmol/L, the Sampson and Friedewald LDL-C values begin to diverge (Supplemental Fig. S2). The difference between Sampson and Friedewald LDL-C values is positively correlated with serum TG level (Supplemental Fig. S3). When TG level < 0.60 mmol/L and non-HDL-C level < 2.50 mmol/L, then on average, the Sampson LDL-C level is less than Friedewald LDL-C level, by 2.8% (95% CI 2.5% to 3.1%; Fig. 2).

Furthermore, the Friedewald LDL-C equation is validated only when TG level is ≤ 4.52 mmol/L, which comprises 98.2% (95% CI 97.7% to 98.6%) of the population. In contrast, the Sampson LDL-C equation can be used for TG levels up to 9.04 mmol/L, which includes 99.9% (95% CI 99.7% to 100%) of the population. For the 1.6% (95% CI 1.3% to 2.1%) of individuals with TG level between 4.52 and 9.04 mmol/L, the Friedewald LDL-C level calculation is unavailable, but the Sampson LDL-C level can be calculated. Their mean Sampson LDL-C level is 2.63 ± 1.07 mmol/L (median 2.53, interquartile range, 2.01 to 3.16 mmol/L).

Clinically, referencing the 2021 Canadian Cardiovascular Society lipid guidelines,^1^ we determined the personal LDL-C treatment threshold for each study participant based on their reported cardiovascular condition(s). Among patients who were taking cholesterol-lowering medications, 29.1% have ASCVD and an LDL-C treatment intensification threshold of 1.8 mmol/L. Most individuals (70.9%) are on cholesterol medications for primary prevention or for a statin-indicated condition, such as diabetes or chronic kidney disease. Their LDL-C treatment intensification threshold is 2.0 mmol/L.

Using personal LDL-C thresholds for participants who reported taking cholesterol-lowering medications, the agreement between the Sampson and Friedewald equations was 96.7% (95% CI 95.1% to 97.8%). Compared to the Friedewald equation, the Sampson equation reclassified LDL-C into a higher category in 3.3% more patients [95% CI 2.2% to 4.9%] and into a lower category for 0% of patients. Applying these results to the estimated 12% of Canadians (3.72 million individuals) who are taking cholesterol-lowering medications, about 123,000 individuals would meet the criteria for treatment intensification after instituting use of the Sampson LDL-C equation in place of the Friedewald equation. Among individuals who were not previously treated with cholesterol-lowering medications and had an LDL-C level < 5 mmol/L, the Sampson equation would reclassify their LDL-C level to 5 mmol/L or higher for 0.34% (95% CI 0.16% to 0.72%) of the general population— that is, an estimated 93,000 Canadians. Conversely, no individuals have a Friedewald-equation LDL-C level ≥ 5 mmol/L and a Sampson-equation LDL-C level < 5 mmol/L.

## Discussion

Our analysis indicates a very strong correlation between the Friedewald and Sampson equations, suggesting that for the large majority of Canadians with relatively normal lipid profiles, a change in equations would lead to minimal to no impact on treatment advice and decisions. Nonetheless, the LDL-C equation change from Friedewald to Sampson/NIH is expected to have a small but tangible impact on cholesterol treatment in Canada. This impact is expected because although Friedewald and Sampson LDL-C values are highly correlated overall, they are divergent for individuals with high TG or very low LDL-C level.

In the first instance, related to hypertriglyceridemia, the potential benefit of using the Sampson equation is more apparent in clinic patients, particularly those with obesity or diabetes. In the past, the laboratory would refrain from reporting Friedewald-equation LDL-C level if TG > 4.52 mmol/L, a level that is uncommon in the general population (perhaps 1 in 50 to 100 Canadians has this level^28^) but is quite commonly encountered in lipid, cardiology, and diabetes clinics. A clear advantage of using the Sampson equation is that valid LDL-C levels can be calculated even when TG levels are as high as 9.04 mmol/L. On the other hand, when TG > 1.5 mmol/L, the Canadian Cardiovascular Society dyslipidemia guidelines recommend using non-HDL-C or apolipoprotein B level as the preferred alternatives to LDL-C level.^1^ This practical alternative makes this advantage of using the Sampson equation less relevant in clinical practice.

In the second instance, related to very low LDL-C levels, this concern was minimal until 2015, when potent LDL-C lowering medications—evolocumab and alirocumab—were approved, the use of which resulted in some patients attaining extremely low LDL-C levels for the first time and at relatively high frequency. Thus, having an equation that is valid and accurate to calculate LDL-C values for patients with low achieved LDL-C levels has become important, and the Sampson equation accomplishes this. But given that the 2021 lipid guidelines do not specify a lower LDL-C level limit for treated patients, the main clinical implication of the Sampson equation is the availability of accurate readings in the very low range of LDL-C values. However, the equation change has potential implications for high-risk patients with treated LDL-C in the vicinity of the intensification LDL-C threshold of 1.80 mmol/L. For instance, on average, in this range, the Sampson equation yields LDL-C values that are slightly higher than those determined by the Friedewald equation. For instance, a Friedewald-equation LDL-C level of 1.80 mmol/L corresponds to a Sampson-equation LDL-C level of 1.86 mmol/L. As a result, some patients who were previously considered to be below the intensification threshold according to Friedewald equation would now exceed the threshold according to the Sampson equation.

Among Canadian patients who report taking cholesterol-lowering medications, after the LDL-C equation is changed from the Friedewald to the Sampson, we estimate that 123,000 more patients (3.3%) may qualify for treatment intensification based on their LDL-C threshold. In addition, among Canadians with a Friedewald-equation LDL-C level < 5 mmol/L and not reported to be taking cholesterol-lowering medications, we estimate that 93,000 individuals (0.34%) would now have a Sampson-equation LDL-C level ≥ 5 mmol/L. These people would thus meet guideline indications for statin treatment. Overall, > 200,000 Canadians might be affected by the LDL-C equation change. Given that the Sampson equation is more accurate than the Friedewald equation, these patients will benefit in the long run from better LDL-C management.

For individuals whose measured LDL-C level is affected by the change from the Friedewald to the Sampson equation—though they might meet indications for cholesterol treatment intensification, whether they would, in practice, receive treatment intensification is unclear. Across the population, the average difference between the Sampson-equation and the Friedewald-equation LDL-C level is only 0.06 mmol/L. This small difference in LDL-C level might be insufficient to overcome therapeutic inertia, especially if an LDL-C level hovering just above the treatment threshold is viewed as good enough to simply continue current management. On the other hand, exceeding the LDL-C treatment threshold only slightly makes cholesterol control an achievable goal for the patient. It presents an opportunity for physicians to control other cardiovascular risk factors more stringently and promote health behaviour interventions that may be sufficient to reduce LDL-C to levels under the guideline thresholds.

Our study has some limitations. First, we used survey data; thus, medical history provided by participants may contain inaccuracies. Furthermore, information on carotid stenosis, abdominal aortic aneurysm, and peripheral vascular disease was unavailable. Second, on the basis that lipid profile distributions were very similar for CHMS vs NHANES, the analysis applied results from NHANES to Canada. Ideally, however, the analysis should be performed using participant-level data in CHMS. Third, for patients who reported taking cholesterol medications, we were unable to distinguish whether they were already on a stable dose or if their healthcare provider was in the process of adjusting the dose.

In our opinion, the Canadian Society of Clinical Chemists’ recommendation to switch from using the Friedewald equation to using the Sampson equation was valid and clinically justified. In the vast majority of patients, this change has no consequence for treatment. Furthermore, for patients with either very high TG or extremely low LDL-C level, this approach has the advantage of obtaining more accurate calculated LDL-C values. However, some patients with LDL-C values in the vicinity of the guideline threshold values may be impacted. Although the proportion of Canadian patients potentially affected is small, we estimate an absolute number of > 200,000 patients who may require reassessment or reassignment of their cholesterol treatment status because of the LDL-C equation change.
