# Supplementary material for: The Impact of Low-Density Lipoprotein Equation Changes on Cholesterol Treatment in Canada
Source: CJC Open. 2022 Oct 1;5(1):37–42. doi: 10.1016/j.cjco.2022.09.007 (PMC9869357; doi:10.1016/j.cjco.2022.09.007)
Supplement: Supplementary Material [file mmc1.pdf]

Supplementary Material

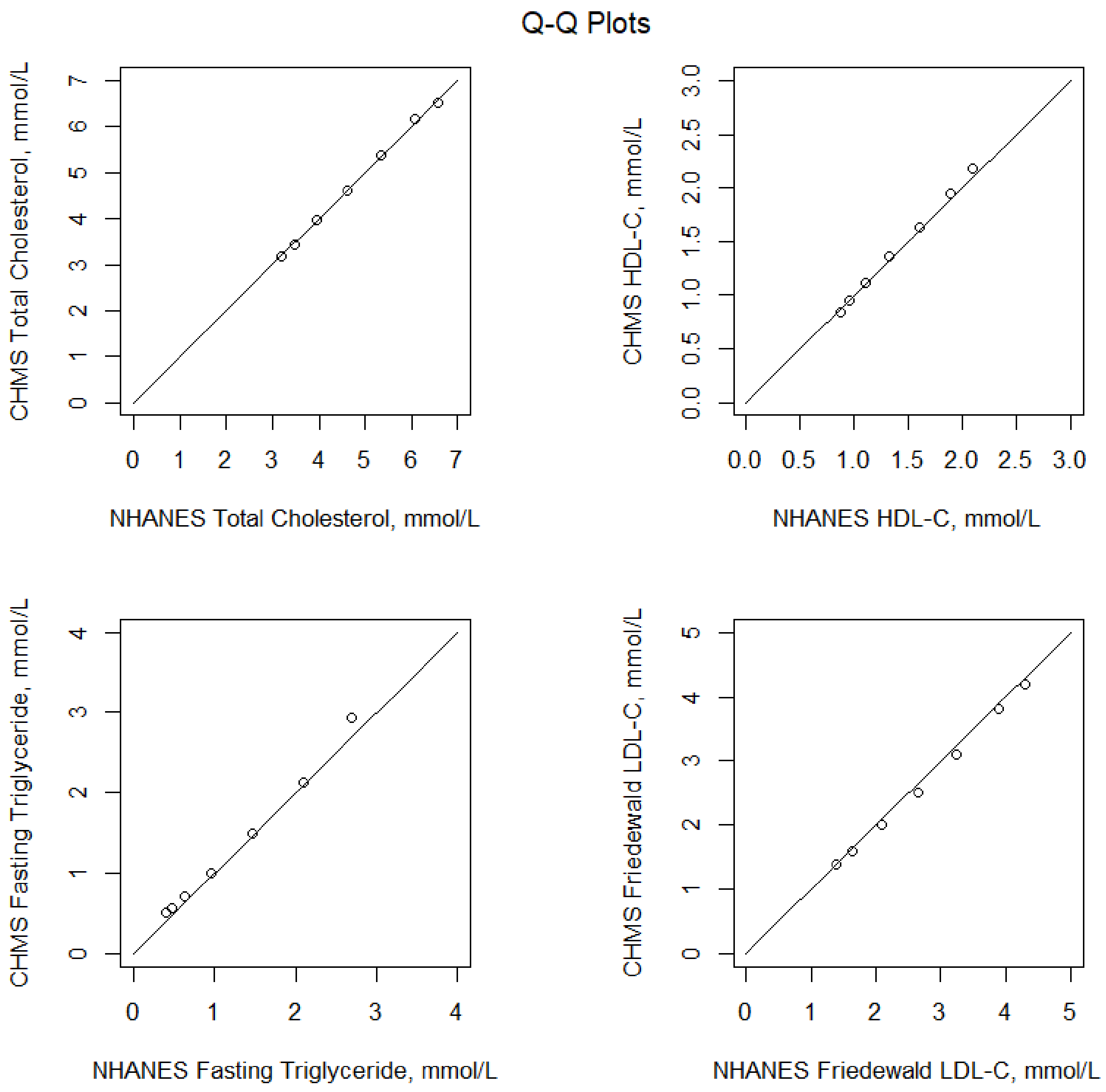

Supplemental Figure S1: Quantile-quantile plots of lipid panel components between the National Health and Nutrition Examination Survey (NHANES) years 2017-2020 and the Canadian Health Measures Survey (CHMS) year 2019. Values are taken at the 5th, 10th, 25th, 50th, 75th, 90th, and 95th percentiles.

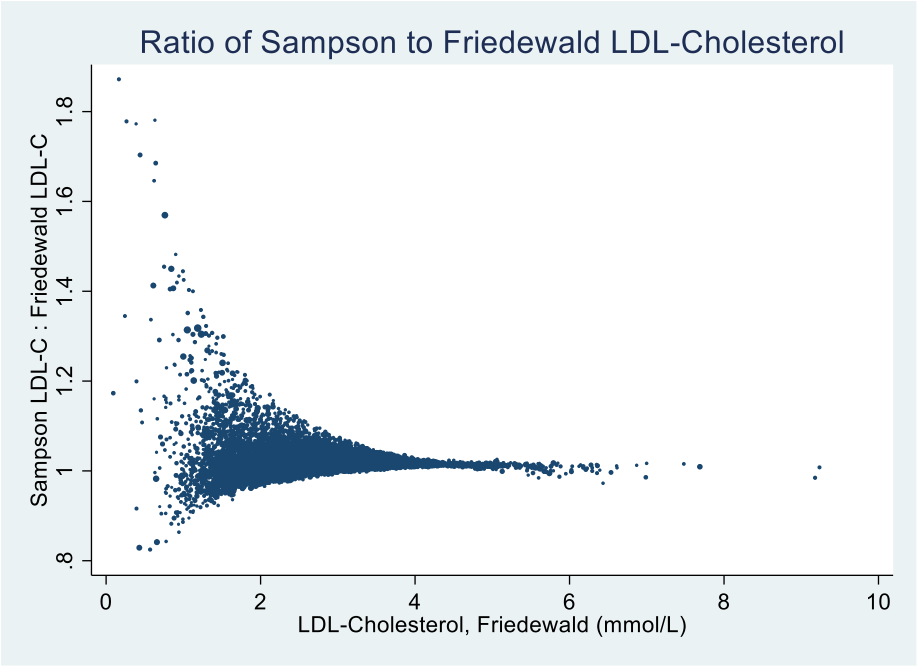

Supplemental Figure S2: Scatterplot of the ratios between Sampson LDL-C to Friedewald LDL-C. For Friedewald LDL-C ≤ 2.5 mmol/L, the LDL-C estimates between the equations diverge, indicating that individuals with low LDL-C are most affected by the choice of equation.

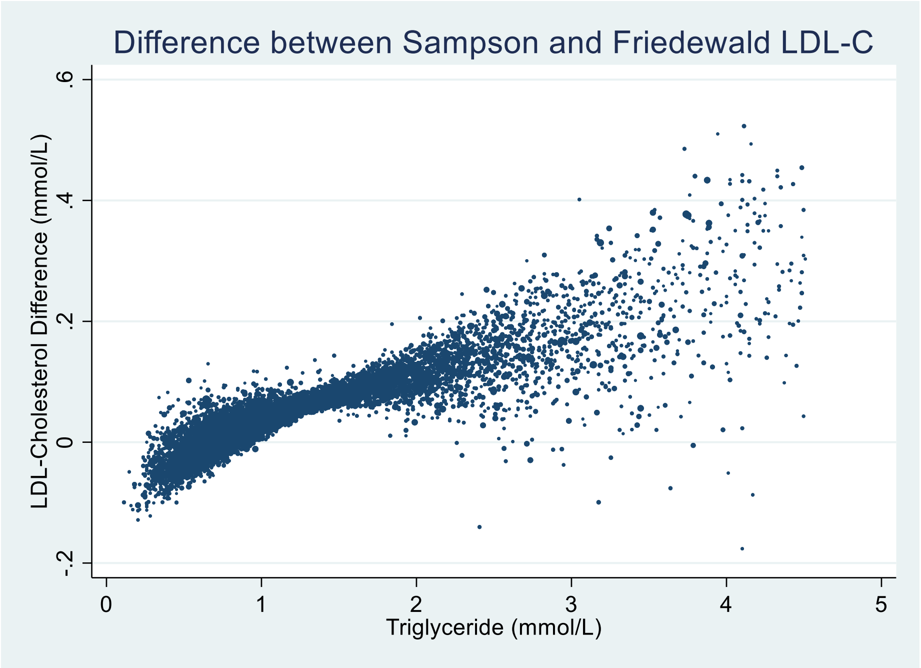

Supplemental Figure S3: Scatterplot of the difference between Sampson and Friedewald LDL-C versus serum triglyceride. When serum triglyceride is elevated, LDL-C tends to be underestimated by the Friedewald equation compared to the Sampson equation.
